# Evaluation of Allocation Schemes of COVID-19 Testing Resources in a Community-Based Door-to-Door Testing Program

**DOI:** 10.1001/jamahealthforum.2021.2260

**Published:** 2021-08-27

**Authors:** Ben Chugg, Lisa Lu, Derek Ouyang, Benjamin Anderson, Raymond Ha, Alexis D’Agostino, Anandi Sujeer, Sarah L. Rudman, Analilia Garcia, Daniel E. Ho

**Affiliations:** 1 Regulation, Evaluation, and Governance Laboratory, Stanford University, Stanford, California; 2 Department of Political Science, Stanford University, Stanford, California; 3 Santa Clara County Public Health Department, San Jose, California; 4 Stanford Institute for Economic Policy Research, Stanford University, Stanford, California; 5 Stanford Institute for Human-Centered Artificial Intelligence, Stanford University, Stanford, California

## Abstract

**Importance:**

Overcoming social barriers to COVID-19 testing is an important issue, especially given the demographic disparities in case incidence rates and testing. Delivering culturally appropriate testing resources using data-driven approaches in partnership with community-based health workers is promising, but little data are available on the design and effect of such interventions.

**Objectives:**

To assess and evaluate a door-to-door COVID-19 testing initiative that allocates visits by community health workers by selecting households in areas with a high number of index cases, by using uncertainty sampling for areas where the positivity rate may be highest, and by relying on local knowledge of the health workers.

**Design, Setting, and Participants:**

This cohort study was performed from December 18, 2020, to February 18, 2021. Community health workers visited households in neighborhoods in East San Jose, California, based on index cases or uncertainty sampling while retaining discretion to use local knowledge to administer tests. The health workers, also known as *promotores de salud* (hereinafter referred to as *promotores*) spent a mean of 4 days a week conducting door-to-door COVID-19 testing during the 2-month study period. All residents of East San Jose were eligible for COVID-19 testing. The *promotores* were selected from the META cooperative (Mujeres Empresarias Tomando Acción [Entrepreneurial Women Taking Action]).

**Interventions:**

The *promotores* observed self-collection of anterior nasal swab samples for SARS-CoV-2 reverse transcriptase–polymerase chain reaction tests.

**Main Outcomes and Measures:**

A determination of whether door-to-door COVID-19 testing was associated with an increase in the overall number of tests conducted, the demographic distribution of the door-to-door tests vs local testing sites, and the difference in positivity rates among the 3 door-to-door allocation strategies.

**Results:**

A total of 785 residents underwent door-to-door testing, and 756 were included in the analysis. Among the 756 individuals undergoing testing (61.1% female; 28.2% aged 45-64 years), door-to-door COVID-19 testing reached different populations than standard public health surveillance, with 87.6% (95% CI, 85.0%-89.8%) being Latinx individuals. The closest available testing site only reached 49.0% (95% CI, 48.3%-49.8%) Latinx individuals. Uncertainty sampling provided the most effective allocation, with a 10.8% (95% CI, 6.8%-16.0%) positivity rate, followed by 6.4% (95% CI, 4.1%-9.4%) for local knowledge, and 2.6% (95% CI, 0.7%-6.6%) for index area selection. The intervention was also associated with increased overall testing capacity by 60% to 90%, depending on the testing protocol.

**Conclusions and Relevance:**

In this cohort study of 785 participants, uncertainty sampling, which has not been used conventionally in public health, showed promising results for allocating testing resources. Community-based door-to-door interventions and leveraging of community knowledge were associated with reduced demographic disparities in testing.

## Key Points

**Question:**

What are effective mechanisms to identify and reach vulnerable populations and equalize access to COVID-19 testing resources in the presence of substantial demographic disparities?

**Findings:**

In this cohort study of 756 participants, a door-to-door program with community-based health workers was associated with a substantial increase in the proportion of Latinx and elderly individuals undergoing testing, relative to neighborhood testing sites. The protocol associated with the greatest increase in testing at-risk individuals was uncertainty sampling, followed by local knowledge, and then targeting households in areas with a high number of index cases.

**Meaning:**

These findings suggest that community-based testing programs that allocate resources using uncertainty sampling might effectively reduce COVID-19 testing disparities.

## Teaser

This cohort study compares 3 protocols for allocating health workers to deliver COVID-19 tests door-to-door within East San Jose, California, including index area selection, uncertainty sampling, and local knowledge of the health workers.

## Introduction

The SARS-CoV-2 pandemic has highlighted long-standing questions about how to allocate testing resources.^1,2,3,4,5,6^ Although random testing is suitable for estimating prevalence,^7,8^ more targeted testing is advocated to reduce transmission of the virus. Such targeting, however, still poses challenges for how to optimally allocate COVID-19 testing resources. The Centers for Disease Control and Prevention recommends testing for symptomatic or exposed individuals^9^; others recommend testing specific at-risk groups, such as frontline health care workers or the elderly,^10,11^ and much discussion focuses on increasing testing resources in vulnerable communities.

In vulnerable communities, little is known about the effectiveness of specific strategies for allocating testing resources. COVID-19 has disproportionately affected low-income and minority populations,^12,13^ in particular the Latinx community.^14,15,16,17^ In Santa Clara County, California, for instance, Latinx individuals constitute less than 26% of the population but accounted for more than 56% of COVID-19 cases at baseline.^18^ This disparity may be due to English language barriers among immigrant and Hispanic populations, distrust of the health care system,^19,20^ housing conditions, essential worker status, and other structural sources. Although increasing the number of diagnostic tests in vulnerable communities is important for reducing health disparities, how to do so is not obvious. COVID-19 testing sites established in such communities continue to exhibit disparities relative to the neighborhood population. It is thus imperative to understand the most effective means to reach subpopulations and allocate testing resources to overcome social and logistical barriers to access.

One increasingly popular method to reach vulnerable populations involves community health care workers known as *promotores de salud* (hereinafter referred to as *promotores*).^21,22,23,24,25^ In Spanish-speaking communities, *promotores* are trusted members of the community who serve as mediators between the health care system and other community members. By lowering language and cultural barriers and providing reliable, trustworthy medical information, *promotores* are able to help disadvantaged communities receive better medical care.^26,27,28^ Indeed, owing to the past success of *promotores*’ efforts, many have advocated involving *promotores* specifically in the COVID-19 response.^29,30,31^ The test-to-care model piloted in San Francisco, California, for example, uses *promotores* to support low-income workers with positive test results for COVID-19.^32^

Although provision of direct service is listed by the US Department of Health and Human Services as 1 of 7 roles of community health workers,^33^ most studies have used *promotores* as translators and health advocates. A core objective of the present study was to assess the effectiveness of *promotores* in delivering COVID-19 tests. Enlisting the help of *promotores* was motivated by observations and focus group insights that formal testing sites—even walk-in sites, with free testing, located in the neighborhood—still did not appear to reach all members of the community. Indeed, despite more than 60% of the population in East San Jose being Latinx, only 30.7% to 49.0% of visitors at the 2 closest testing sites were Latinx (see the Results section).

In this report, we compare 3 protocols for allocating *promotores* to directly engage community members at home and deliver tests. The first protocol, index area selection, assigns workers to areas with a high number of confirmed COVID-19 cases per capita. This approach is suggested by the existing disease surveillance literature, which prioritizes testing individuals who have been in contact with infected individuals to increase efficacy (higher likelihood of finding new infections) and efficiency (fewer overall tests required).^34,35,36,37^ The second protocol, uncertainty sampling (ie, upper confidence bound sampling), is borrowed from sequential decision-making in machine learning and selects neighborhoods with the highest potential positivity rate, accounting for uncertainty.^38^ The third protocol uses *promotores*’ local knowledge to identify where testing is needed.

## Methods

This study followed the Strengthening the Reporting of Observational Studies in Epidemiology (STROBE) reporting guidelines for cross-sectional studies. The Santa Clara County Public Health Department and Stanford University, Stanford, California, deemed the work public health surveillance; the Revised Common Rule deems “public health surveillance activities,” including testing necessary to monitor disease outbreaks by a public health authority, not subject to IRB [institutional review board] oversight under 45 CFR §46. Participation in this study was voluntary: COVID-19 testing was undertaken only when permission was granted, and the *promotores* volunteered for the role.

### Data Source and Measurement

We used the California Reportable Disease Information Exchange (CalREDIE) database,^39^ which contained results for all COVID-19 tests administered in the State of California. We also obtained direct access to laboratory results from the facility that processed all door-to-door tests administered by the *promotores* and from 2 other proximate testing sites: Emmanuel Baptist Church and the Santa Clara County Fairgrounds. Direct laboratory results contain more comprehensive demographic information than CalREDIE, allowing us to compare population differences between the *promotores*’ visits and these other testing sites. Demographic information, including race and ethnicity, was self-reported by individuals and was important given the study objective.

### Setting

**Figure 1.**
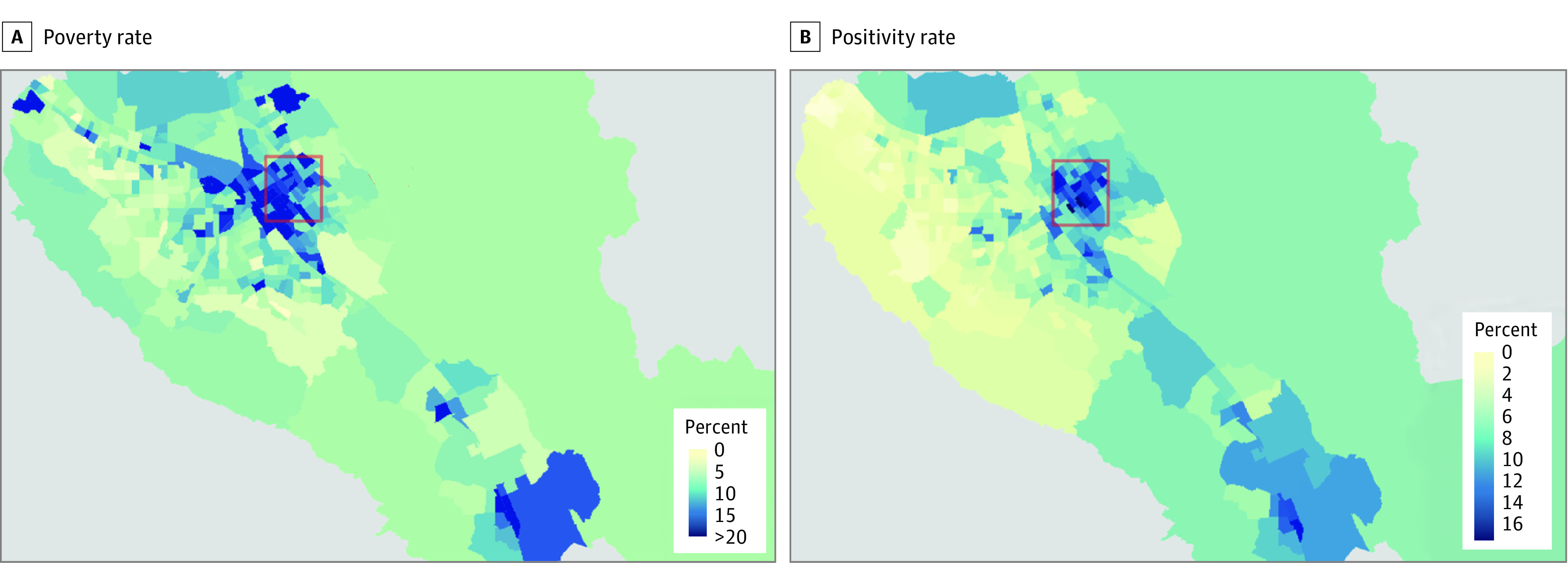
Poverty and COVID-19 Positivity Rates in Santa Clara County, California A, The poverty rate is calculated as percentage of residents living below the poverty line, from a Santa Clara County Public Health Department report from the 2015 American Community Survey. B, The positivity rate is calculated as the percentage of all conclusive tests returning positive results at the census tract level from the California Reportable Disease Information Exchange database. Both rates were aggregated from November 18 to December 18, 2020, 4 weeks before the intervention began. The red rectangle indicates the area of East San Jose in which the intervention took place.

Our intervention was designed for East San Jose in Santa Clara County, one of the top 20 most populous US counties with approximately 1.9 million residents. Figure 1A plots the poverty rate across census tracts in the county, with East San Jose denoted by the red box. Figure 1B plots the positivity rate (the percentage of all conclusive tests returning positive results) at baseline. Positivity rates were highest in East San Jose, and there is a striking correlation between poverty and positivity rates, which animated our community-based intervention. The field trial ran from December 18, 2020, to February 18, 2021, in the 3 primary zip codes constituting East San Jose (95122, 95116, and 95127). In addition to the poverty difference, this area has lower levels of educational attainment (31.3% of residents without a high school degree) and English proficiency (38.2% of residents speak English less than very well) and a much higher proportion of Latinx residents (60.2% of residents) than Santa Clara County overall (see Table 1). In the 4 weeks before our intervention, these 3 zip codes had both a positivity rate (13.1%) and case rate (percentage of all positive test results relative to population size [9500 per 100 000 population]) more than double that of the county (6.4% and 4500 per 100 000 population, respectively).

**Table 1.** Study Population Disparities Between Santa Clara County and East San Jose, California

Characteristic	Study population^a^	
	Santa Clara County (n = 1 927 470)	East San Jose (n = 178 202)
COVID-19		
Baseline positivity rate^b^	6.4	13.1
Baseline case rate^c^	4500	9500
Demographic data		
Latinx ethnicity	25.5	60.2
Low English proficiency^d^	20.2	38.2
Below poverty line	7.5	10.7
No high school diploma	11.6	31.3
Foreign born	39.2	44.8
Sex		
Male	50.6	51.4
Female	49.5	48.6
Age, y		
≥65	13.2	11.6
≤17	22.2	25.0

^a^ Unless otherwise indicated, data are expressed as percentage of individuals. Demographic and total population data are from the 2019 American Community Survey 5-year estimates; COVID-19 testing and case-rate statistics, from the California Reportable Disease Information Exchange (CalREDIE).^39^

^b^ Calculated as the percentage of tests that yielded a positive test result.

^c^ Calculated as the number of cases per 100 000 residents in the 4 weeks before the intervention.

^d^ Measured by whether the respondent speaks English less than very well.

### Participants

The *promotores* were selected from the META cooperative, an acronym for Mujeres Empresarias Tomando Acción (Entrepreneurial Women Taking Action), which consists of active community leaders in East San Jose. They were trained by the Santa Clara County Public Health Department to offer and observe self-collection of COVID-19 tests (anterior nasal swabs) and deliver the specimens to a collection site at the end of each day. The *promotores* were paid as contractors and received health care subsidies. On visiting a household in pairs, they recorded information about the visit on county-provided mobile devices in databases accessible by the Stanford University team. All individuals in the target area were eligible for testing.

### Study Design

We examined 3 COVID-19 testing protocols: index area selection, uncertainty sampling, and local knowledge. Index area selection is based on the same principle as contact tracing, namely, to intervene where an index case can pose risk of (secondary) infection. The motivating assumption is that unidentified individuals with infection are likely to be among contacts of individuals identified with COVID-19 infection.^37^ We therefore chose segments with the largest number of positive COVID-19 test results per capita during the past day.

Uncertainty sampling attempts to target the principal measure of inadequate testing resources, namely, the positivity rate. Uncertainty sampling is different from merely targeting the positivity rate, however, because it accounts for the uncertainty in the rate.^40^ This is important because underserved communities may have a seemingly low positivity rate owing to randomness and low testing volume. By itself, a low positivity rate is not sufficient for public health authorities to rule out much higher positivity rates. Indeed, high uncertainty may reflect marginalization and isolation from services. We thus borrow from the machine learning literature and chose segments based on the upper confidence bound of the mean positivity rate (number of positive tests divided by the number of total tests) of census tracts during the previous 7 days. Confidence bounds were calculated as 95% binomial proportion CIs, where the number of successes corresponds to the number of positive tests. See eFigure 1 in the Supplement for an illustration of the upper confidence bounds on the positivity rate, and eFigure 2 in the Supplement for a demonstration of the efficacy of upper confidence bound sampling versus naive prevalence sampling.

In designing the intervention with stakeholders, the *promotores* expressed a desire to use local knowledge (eg, awareness of a social gathering) in allocating COVID-19 testing resources. We therefore designed the intervention to dedicate 2 days per week for *promotores* to use their own judgment as to where to conduct testing.

We collectively refer to uncertainty sampling and index area selection as *risk selection*. If there were *N* teams available for risk selection, the top *2N* segments were chosen. Half of these were randomly assigned to the *promotores* and the other half were withheld as control segments for comparison. Although the *promotores* did not visit these control segments, individuals in those segments may still have been tested by other means (eg, local testing sites). Typically, visits were conducted 4 days of the week (Tuesday, Wednesday, Thursday, and Sunday); 2 of the 4 days were dedicated to risk selection and the other 2 were dedicated to local knowledge. On days dedicated to risk selection, each available team of *promotores* was randomly assigned 1 of the 2 selection strategies, with a maximum of 2 teams per strategy.

### Neighborhoods

To reduce travel time, the area was divided into small, walkable, contiguous zones, which we term *segments* (eMethods in the Supplement). Segments were generated by first dividing the area into its census tracts contained in the 3 zip codes of interest. Based on address lists (using Melissa Inc data^41^), census tracts were then further divided into groups of approximately 100 households along a compact route, as displayed in eFigure 3 in the Supplement.

Each morning, teams were provided a map and list of addresses on a mobile device. The devices were used for navigation and data entry in the field. The *promotores* finalized data entry on their return to the office in the afternoon. More details about data and sample collection can be found in eFigure 4 in the Supplement.

### Variables

We examined our results along 3 dimensions: (1) whether the program increased COVID-19 testing capacity (overall number of tests conducted), (2) whether the program reduced demographic testing gaps, and (3) which allocation scheme was most effective at identifying risk (defined as those with positive test results for COVID-19). With regard to dimension 1, a key question was whether individuals would be willing to secure COVID-19 tests from a door-to-door exchange.

Outcome variables of interest from the door-to-door intervention were (1) where and whether a test was conducted, (2) demographic attributes of those undergoing testing (sex, ethnicity, race, and age), and (3) the result of the test. We also investigated the response rates (the number of tests conducted as a fraction of the number of people visited) of the 3 protocols.

### Statistical Analysis

Analyses involved recording and observing the frequency of positive test results and the distribution of demographic attributes by testing strategy. All *P* values and exact 95% CIs for demographic differences and positive rates were calculated using χ^2^ tests. Two-sided *P* < .05 indicated statistical significance. All data were processed and results were calculated in R, version 3.6.2 (R Program for Statistical Computing).

## Results

The *promotores* conducted 785 total tests in East San Jose. We discarded 29 tests that were performed for nonresidents or could not be reliably matched to assigned households, leaving 756 test results available for the analysis (female, 61.1% [95% CI, 57.5%-64.6%]; male, 37.2% [95% CI, 33.7%-40.1%]; other or unknown sex, 1.7% [95% CI, 0.9%-2.9%]; aged 45-64 years, 28.2% [95% CI, 25.6%-32.2%]).

### Testing Capacity

**Figure 2.**
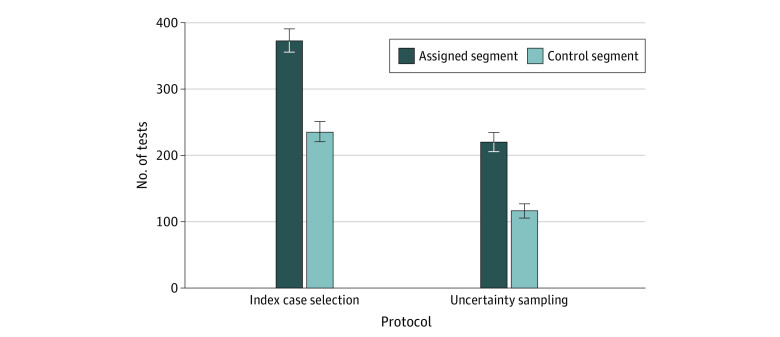
Total Number of Tests Conducted in Assigned and Control Segments The number of tests conducted by the community health workers (*promotores*) from the laboratory data is restricted to risk selection (tests based on local knowledge are not shown). The 95% CIs in the error bars are based on SEs.

Because individuals may secure COVID-19 testing from other sources, we examined whether door-to-door testing provided a net increase in testing capacity in the subset of risk-selected tests. We did so by comparing the test volume of assigned segments against the (randomized) control segments using CalREDIE data. Figure 2 presents the volume of tests in the assigned and control segments during our observation window. Test volume increased with the assigned compared with control segments for both uncertainty sampling (217 [95% CI, 202.6-231.4] vs 114 [95% CI, 103.5-124.5] tests) and index area selection (373 [95% CI, 354.1-391.9] vs 233 [95% CI, 218.0-248.0] tests). This represents a 90% increase in segments chosen by uncertainty sampling, and a 60% increase in those chosen by the index area protocol.

### Testing Disparities

We compare demographic attributes for individuals undergoing testing during the same period at 2 proximate testing sites: Emmanuel Baptist Church and the Santa Clara County Fairgrounds. The tests at the church were self-administered nasal swabs (observed by health care professionals), whereas those at the fairgrounds were administered by health care professionals. Testing at the fairgrounds site was by appointment only and was open 7 days a week. The church had drop-in COVID-19 testing and was open Tuesday through Friday every week.

Across 4 categories—sex, ethnicity, race, and age—the distribution of door-to-door test recipients was significantly different than those of the 2 testing sites (Table 2). Whereas 49.0% (95% CI, 48.3%-49.8%) of individuals at the church and 30.7% (95% CI, 30.5%-30.9%) of individuals at the fairgrounds were Latinx, 87.6% (95% CI, 85.0%-89.8%) of those undergoing testing via the door-to-door intervention were Latinx (*P =* .001), representing an 80% to 184% relative increase in Latinx individuals reached. Individuals also declined to report race at much higher rates in the testing sites (fairgrounds, 16.1% [95% CI, 13.8%-16.2%]; church, 27.8% [95% CI, 27.2%-28.5%]) than in the door-to-door tests (7.9% [95% CI, 6.1%-10.1%]), possibly indicating greater trust in the *promotores* (*P* = .001). The door-to-door intervention tested 61.1% women (95% CI, 57.5%-64.6%) compared with 50.5% (95% CI, 49.7%-51.2%) for the church and 51.9% (95% CI, 51.7%-52.1%) for the fairgrounds (*P* ≤ .006). Door-to-door tests also reached 12.7% (95% CI, 10.4%-15.3%) of individuals 65 years or older compared with 7.8% (95% CI, 7.4%-8.2%) at the church and 5.4% (95% CI, 5.3%-5.6%) at the fairgrounds (*P* = .001). Demographic differences within the 3 door-to-door protocols are provided in the eTable in the Supplement.

**Table 2.** Demographic Differences Between Testing Sites and Door-to-Door Testing

Characteristic	Proportion, % (95% binomial CI)^a^			*P* value^b^	
	Fairgrounds	Church	Door-to-door	Door-to-door vs fairgrounds	Door-to-door vs church
Sex					
Female	51.9 (51.7-52.1)	50.5 (49.7-51.2)	61.1 (57.5-64.6)	.006	.002
Male	45.1 (44.9-45.4)	46.6 (45.9-47.3)	37.2 (33.7-40.1)	.006	.002
Unknown/other	3.0 (2.9-3.0)	2.9 (2.8-3.2)	1.7 (0.9-2.9)	.07	.08
Ethnicity					
Latinx	30.7 (30.5-30.9)	49.0 (48.3-49.8)	87.6 (85.0-89.8)	.001	.001
Not Latinx	41.6 (41.4-41.8)	23.2 (22.6-23.8)	1.5 (0.7-2.6)	.001	.001
Other/declined^c^	27.7 (27.5-27.9)	27.8 (27.1-28.4)	11.0 (8.8-13.4)	.001	.001
Race					
White	21.9 (21.7-22.1)	13.2 (12.7-13.7)	27.5 (24.4-30.8)	.003	.001
Asian	38.5 (38.2-38.7)	32.7 (32.0-33.4)	8.6 (6.7-10.8)	.001	.001
Black	1.7 (1.6-1.8)	2.9 (2.7-3.1)	0 (0-0.1)	.008	.001
Not reported	16.1 (15.9-16.2)	27.8 (27.2-28.5)	7.9 (6.1-10.1)	.001	.001
Multirace/other^d^	19.5 (19.3-19.7)	21.0 (20.4-21.6)	55.6 (51.9-59.1)	.001	.001
Age, y					
0-17	9.7 (9.5-9.8)	13.0 (12.5-13.5)	20.6 (17.8-23.7)	.001	.001
18-24	19.4 (19.2-19.5)	18.2 (17.6-18.7)	10.1 (8.0-12.4)	.001	.001
25-44	42.4 (42.2-42.7)	32.4 (31.7-33.1)	27.8 (24.6-31.1)	.001	.06
45-64	23.1 (22.9-23.3)	28.6 (28.0-29.3)	28.2 (25.6-32.2)	.005	.96
≥65	5.4 (5.3-5.6)	7.8 (7.4-8.2)	12.7 (10.4-15.3)	.001	.001

^a^ Testing sites were Emmanuel Baptist Church and the Santa Clara County Fairgrounds. Sample sizes were 164 977 for the fairgrounds, 18 236 for the church, and 756 for door-to-door.

^b^ Indicates results from pairwise comparisons between the door-to-door demographics and fairground demographics or church demographics using a χ^2^ test.

^c^ Individuals in this category indicated prefer not to state or other.

^d^ American Indian or Alaska Native and Pacific Islander racial and ethnic categories are omitted owing to a low baseline rate.

### Effectiveness of Allocation Schemes

The overall positivity rate for door-to-door tests was 6.8% (95% CI, 5.0%-8.9%). It was 10.8% (95% CI, 6.8%-16.0%) for uncertainty sampling, 2.6% (95% CI, 0.7%-6.6%) for index area selection, and 6.4% for local knowledge (95% CI, 4.1%-9.4%) (*P* < .001) (Table 3). By comparison, the positivity rate at the church was 10.1% (95% CI, 9.7%-10.6%) and at the fairgrounds was 8.1% (95% CI, 8.0%-8.3%), likely reflecting more symptomatic testing.

**Table 3.** COVID-19 Positivity Rates of Door-to-Door Protocols

Protocol	Positivity rate, % (95% binomial CI)	No. of tests^a^
Uncertainty sampling	10.8 (6.8-16.0)	195
Index area selection	2.6 (0.7-6.6)	153
Local knowledge	6.4 (4.1-9.4)	361

^a^ Includes those resulting in either positive or negative results (as opposed to inconclusive or invalid results).

The response rate, defined as the fraction of visits resulting in at least 1 test, was also different for the 3 methods. It was highest for local knowledge selection at 50.5% (95% CI, 46.0%-55.0%), followed by 23.4% (95% CI, 20.2%-28.4%) for uncertainty sampling and 10.7% (95% CI, 8.5%-13.3%) for index area selection (*P* < .001). The low response rate for index area selection suggests that households with index cases may either be less likely to answer the door, perhaps to avoid potential exposure, and/or have prompted other household members to get tested, thereby obviating the need for additional COVID-19 testing.

## Discussion

In this community-based intervention with community health workers, we found that allocating COVID-19 tests using machine learning can increase testing capacity, reduce demographic disparities in testing, and detect clusters of infected individuals. Our results demonstrate one effective, data-driven method to improve equity in COVID-19 testing. Contrary to the strategy suggested by contact tracing, testing based on a single measure of incidence (index area selection) may not be as efficient as testing using uncertainty sampling or local knowledge by community-based *promotores*.

Testing based on local knowledge poses a tradeoff with uncertainty sampling. On the one hand, uncertainty sampling displayed a higher positivity rate; on the other hand, local knowledge selection showed a higher response rate. Where a network of *promotores* with local knowledge is unavailable, however, existing public health surveillance data can be used to allocate testing resources to neighborhoods with high uncertainty about the positivity rate.

We found that door-to-door COVID-19 testing was associated with increased testing capacity and an increased ability to reach particular demographic groups. We also found an association between community-based strategies and an extension of disease surveillance to reduce demographic gaps, potentially reducing language barriers, distrust of public health authorities, and transaction costs to getting tested. Such strategies may be more resource intensive, but conventional testing approaches—including neighborhood testing sites—do not appear to reach the most vulnerable members of the community compared with a door-to-door strategy.

Our results point to a capacity-equity tradeoff in testing strategies: high-volume testing sites that benefit from economies of scale are beneficial for reaching large proportions of a community, but lower-volume testing strategies that are more resource intensive can help reduce demographic gaps. The door-to-door intervention had dramatic effects, increasing the proportion of Latinx individuals undergoing testing by 80% to 184% and providing testing for residents who otherwise would not have had any. On a case-by-case basis, public health officials will therefore want to assess the operational costs of high-volume testing sites and the equity benefits of community-based interventions. A full cost-effectiveness analysis remains beyond the scope of the present study. Regardless, our trial demonstrates that the *promotores* model, combined with insights from machine learning, produces unique, complementary benefits and may be of substantial promise in public health.

These results have important implications for public health and addressing the deep social disparities of COVID-19. The protocols are currently being extended for improving vaccine administration in areas characterized by vaccine hesitancy. This approach may also have potential for a range of other public health interventions and testing, such as surveillance for lead exposure among children and testing for other communicable diseases.

### Limitations

There are several limitations to consider when interpreting our results. First, we were unable to draw clean causal inferences about the effect of the intervention on incidence rates. This is primarily because the intervention itself consists of testing, which alters the composition of who undergoes testing. Second, because randomization occurred for risk-selected segments, we cannot assess the effect on testing capacity of local knowledge selection, because we have no geographic comparison group. Third, because the team of *promotores* has deep roots in the community, generalizability to other communities lacking such a network is less clear.

## Conclusions

The results of this cohort study suggest that delivering COVID-19 tests with community health workers, in conjunction with machine learning–based allocation strategies, may help reduce demographic disparities in testing and identify clusters of individuals with high positivity rates. Future work should examine the effectiveness of this approach in other elements of pandemic response, such as vaccination campaigns, as well as other public health areas.

## Notes

Supplement. **eMethods.** Definitions and Procedures **eFigure 1.** Positivity Rate and Upper Confidence Bounds for Census Tracts **eFigure 2.** Surplus of Positive Tests Caught by Upper Confidence Bound Sampling (UCB) **eFigure 3.** Segments Where Intervention Was Implemented **eFigure 4.** Overview of the Workflow **eTable.** Demographic Breakdown of the 3 Door-to-Door Intervention Strategies
eReferences
