# Supplementary material for: Evaluation of Allocation Schemes of COVID-19 Testing Resources in a Community-Based Door-to-Door Testing Program
Source: JAMA Health Forum. 2021 Aug 27;2(8):e212260. doi: 10.1001/jamahealthforum.2021.2260 (PMC8796878; doi:10.1001/jamahealthforum.2021.2260)
Supplement: Supplement. — eMethods. Definitions and Procedures eFigure 1. Positivity Rate and Upper Confidence Bounds for Census Tracts eFigure 2. Surplus of Positive Tests Caught by Upper Confidence Bound Sampling (UCB) eFigure 3. Segments Where Intervention Was Implemented eFigure 4. Overview of the Workflow eTable. Demographic Breakdown of the 3 Door-to-Door Intervention Strategies eReferences [file jamahealthforum-e212260-s001.pdf]

Supplementary Online Content

Chugg B, Lu L, Ouyang D, et al. Evaluation of allocation schemes of COVID-19 testing resources in a community-based door-to-door testing program. *JAMA Health Forum*. 2021;2(8):e212260. doi:10.1001/jamahealthforum.2021.2260

eMethods. Definitions and Procedures

eFigure 1. Positivity Rate and Upper Confidence Bounds for Census Tracts

eFigure 2. Surplus of Positive Tests Caught by Upper Confidence Bound Sampling

(UCB)

eFigure 3. Segments Where Intervention Was Implemented

eFigure 4. Overview of the Workflow

eTable. Demographic Breakdown of the 3 Door-to-Door Intervention Strategies

eReferences

This supplemental material has been provided by the authors to give readers additional information about their work.

© 2021 Chugg B et al, *JAMA Health Forum*.

eMethods. Definitions and Procedures

Segment Definitions
There are 20 census tracts contained in the three ZIP codes 95122, 95116, and 95127. Each census tract was refined into segments: smaller, contiguous, geographic units that the promotores would be able to cover in one day. Segments were created with an algorithm that grouped parcels with matching street names, then split longer streets while combining shorter adjacent streets, and iteratively aimed for a compact arrangement of approximately 100 households, accounting for multifamily buildings. eFigure 1 illustrates the segments and situates where the intervention was implemented.

Field Details and Data Entry
The door-to-door testing program involved collaboration between a research team at Stanford, Case Investigation and Contact Tracing (CICT) teams at the Santa Clara County Department of Public Health, a large testing laboratory, and the teams of promotores from META (Mujeres Empresarias Tomando Acción). Key steps in the information and data flow can be categorized into three phases as shown in eFigure 2.

Before the Visit Through a data sharing agreement with the County, the Stanford team gained secure access to daily COVID-19 testing data from the CalREDIE state database. Each day before a given field assignment, the Stanford team processed the most recent seven days of positive case counts to identify recent index cases (for the index area selection protocol) and calculate the upper confidence bound positivity rate (for the uncertainty sampling protocol) for each census tract (see eFigure 3). From the top census tracts based on recent tests, the Stanford team generated assigned segments, which were sent to the promotores for use the following day, and control segments, which were retained for future comparative analysis. The assigned segments were provided as CSV files with supplementary maps as HTML files through a secure platform. County staff then imported the CSV files into Excel workbooks that would be accessible by the promotores in the field through secure, County-issued mobile devices, with each pair having a specific segment's worth of addresses and fields of information to complete for each address.

Door-to-Door House Visits The promotores teams traveled to their assigned segment for the day and visited as many addresses as they could manage within a single shift, typically 3 hours. At each address, using the editable fields in the Excel workbook, the teams indicated whether they were able to visit the house and knock on the door or ring the doorbell, then whether they were able to have a conversation with a resident at the address. After following a standard script and protocol for offering and administering testing, the promotores indicated on the workbook the number of tests they collected from the address, which was later used as a cross-check with lab data, and any miscellaneous notes. In addition to visiting the Stanford-assigned addresses, the promotores had the discretion to also visit other addresses during their shift, which they could enter into the same workbook, and which would be identifiable in the data analysis as addresses generated through local knowledge.

After the Visit At the end of the daily shift, the promotores teams dropped off physical testing samples at a local collection site, after which the samples were delivered to and tested by the laboratory, and the resulting data was routed to the CalREDIE system. Through an arrangement with the lab, the data for the tests collected by the promotores was also flagged and directly routed to specialized CICT teams, so that the residents who interfaced with the door-to-door program could receive high-touch support, and to the Stanford team for detailed analysis. The laboratory also provided data for the Church and Fairgrounds testing sites, and this dataset included demographic data about testing site visitors which is not otherwise available from CalREDIE. The promotores teams and County staff also cleaned up and finalized the Excel workbook entries at the end of each day and transferred the data back to the Stanford team. Given the identification of assigned and control segments for a specific day, the CalREDIE system was used a few days later to identify segment-level testing and positivity rates.

Protocol Details
We provide more formal definitions of the risk-based protocols, as well as some simulations to illustrate when uncertainty sampling provides reliable gains over naive prevalence sampling.

For both protocols, once assigned (visited) and control segments were chosen for a day, they were removed from consideration for the following two weeks. After that, they were re-eligible for selection. A two week period was chosen based on the incubation period for COVID-19.[1]

© 2021 Chugg B et al. *JAMA Health Forum.*

Index Area Selection Let I_s be the number of index cases in segment *s* over the past day, and N_s its population size. Index area selection was made by ordering segments according to the quantity I_s/N_s and choosing either the top two or four (depending on whether there were one or two teams). We then randomized the selected segments, withholding half as a control set.

Uncertainty Sampling For a segment tract *i* let $\alpha_i$and β_i be the total number of positive and negative COVID-19 tests *over the past seven days* in accordance with County practice. We do not consider inconclusive tests. The positivity rate is:

$$\lambda_i \ = \ \alpha_i \,/\, (\alpha_i \ + \beta_i).$$

The upper confidence bound is calculated as the upper bound of the Clopper-Pearson 95% confidence interval[2], i.e., the maximum (supremum) $\theta_i$such that

$$P(Bin(\alpha_i + \beta_i, \theta_i) \ \geq \alpha_i) \ > \ 0.95 \,/\, 2,$$

where $Bin(n, \theta)$is a Binomial random variable with *n* trials and success probabilityθ.Once the tract(s) with the highest upper confidence bound is chosen, we randomly select two segments from within the tract: one to visit and one to use as a control. The upper confidence bound is a function of both the underlying infection rate in the population tested as well as the number of tests, due to sampling variability. eFigure 3, for instance, illustrates the positivity rate and upper confidence bound in census tracts for a specific period of time. While the point estimate of census tract 3 is higher than that of tract 4, for instance, the low volume in the former means that we have more uncertainty about how high the rate might in fact be. This means that we would allocate more testing resources to the latter.

Next, we perform a Monte Carlo simulation to demonstrate the benefits of uncertainty sampling when prevalence rates and testing rates may be only semi-correlated or even uncorrelated. We generate k = 20 areas, draw vectors of prevalence rates and testing rates uniformly at random (independently of one another). We calculate the resulting positivity rate from naive sampling (allocating tests to where the point estimate is highest) and uncertainty sampling. eFigure 4 summarizes the results from 5000 simulations, each with 100 simulated COVID-19 tests.

These simulations show that high variance in testing and prevalence rates result in the greatest performance gains from uncertainty sampling. Moreover, we see that uncertainty sampling never performs worse than prevalence sampling, unless the testing rates and prevalence rates are utterly correlated. The contrary conditions are, of course, precisely what motivated this allocation protocol: Santa Clara County was facing substantial disparities in case prevalence rates and the testing intervention was a response to the perceived inadequacies of testing resources in vulnerable communities. We hence believe that those are two critical factors for applying this intervention in other areas.

Demographic Differences within Door-to-Door Protocols

While there are sharp demographic differences between door-to-door testing and conventional testing sites, the demographic differences within the three selection protocols are more muted (see eTable 1). The local knowledge protocol tested a higher percentage of Latinx individuals than risk selection (94% compared to 78% and 85%, *p*-value < 0.001, χ^2 test). Both risk selection strategies also seemed to test older individuals (65+) at higher rates than local knowledge — 15% and 19% compared to 9% (*p*-value = 0.02, χ^2 test). Regardless of selection protocol, door-to-door testing reached more Latinx, women, and elderly individuals.

© 2021 Chugg B et al. *JAMA Health Forum.*

eFigure 1. Positivity Rate and Upper Confidence Bounds for Census Tracts

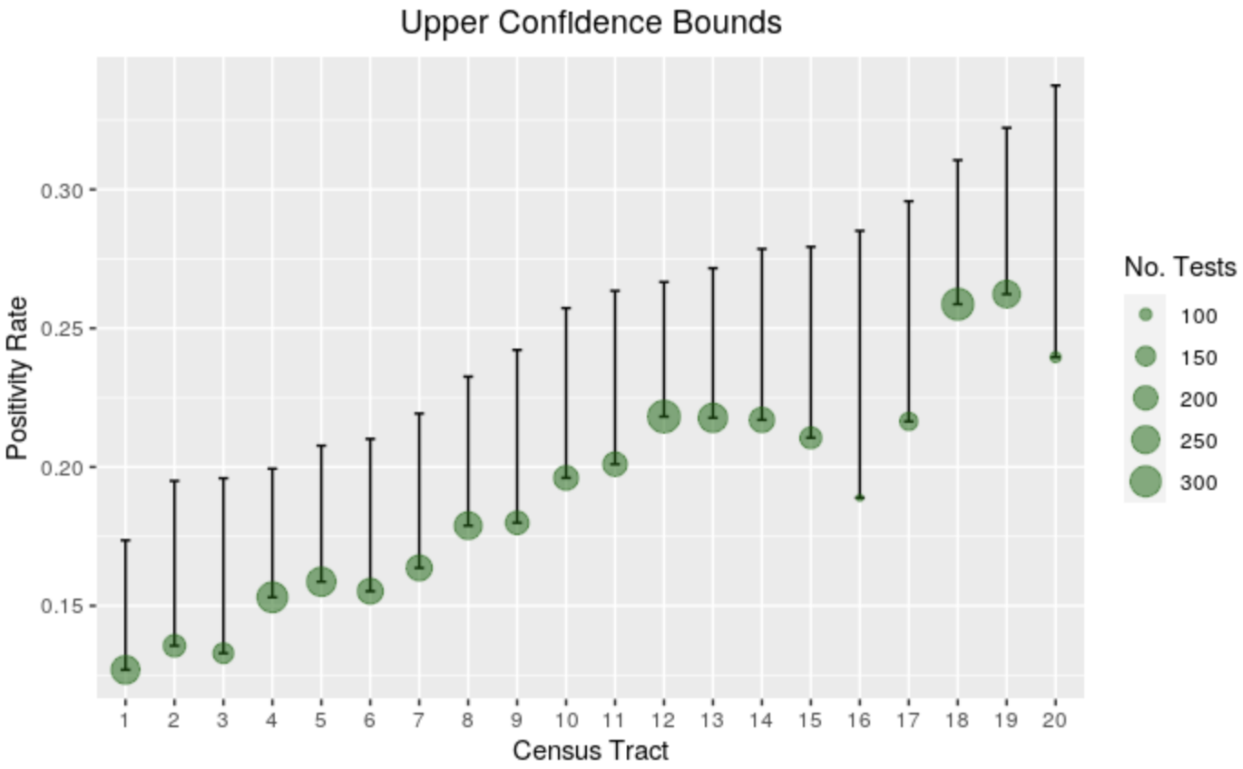

Positivity rates (circles) with upper confidence bounds (bars) for the census tracts under consideration. Census tracts are ordered by their upper confidence bound. The sizes of the points indicate the number of tests in that tract over the past seven days. While the positivity rates are correlated with the upper confidence bounds, there are instances where a tract with a lower positivity rate than its neighbor nevertheless has a higher upper confidence bound. For instance, tract 20 has the highest upper confidence bound despite not having the highest positivity rate. These data were computed for the assignments on January 4th.

© 2021 Chugg B et al, *JAMA Health Forum*.

eFigure 2. Surplus of Positive Tests Caught by Upper Confidence Bound Sampling (UCB)

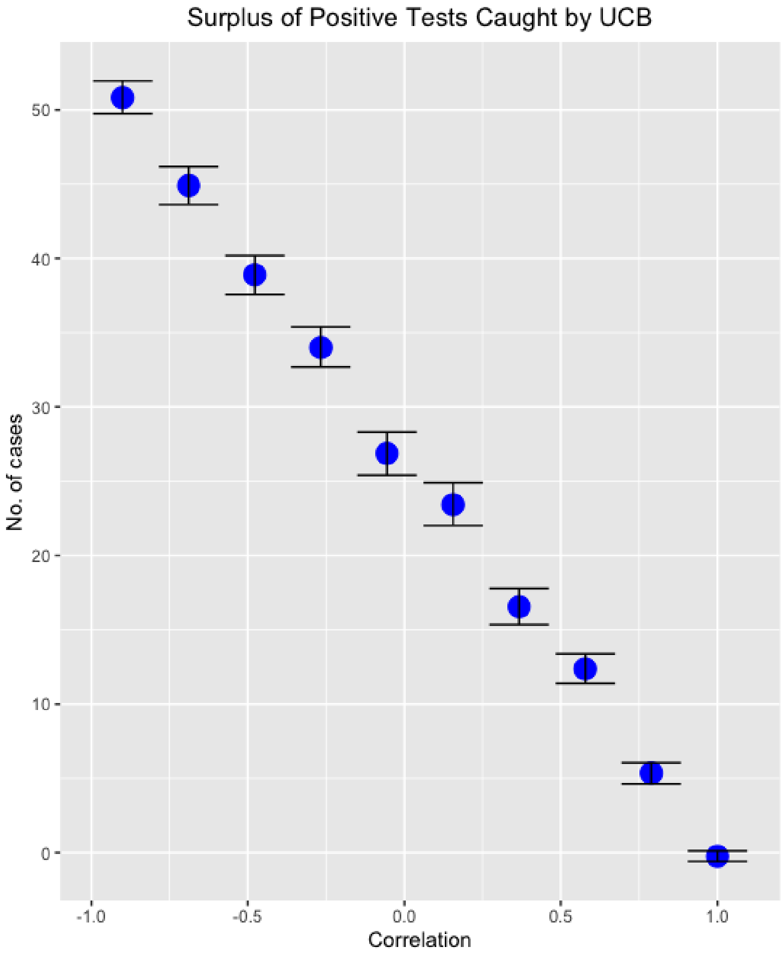

Performance of upper confidence bound sampling (UCB) versus naive prevalence sampling. The *x*-axis is the correlation between the vector of prevalence rates and that of testing rates and the *y*-axis is the surplus of cases detected from UCB versus naive prevalence sampling.

© 2021 Chugg B et al. *JAMA Health Forum.*

eFigure 3. Segments Where Intervention Was Implemented

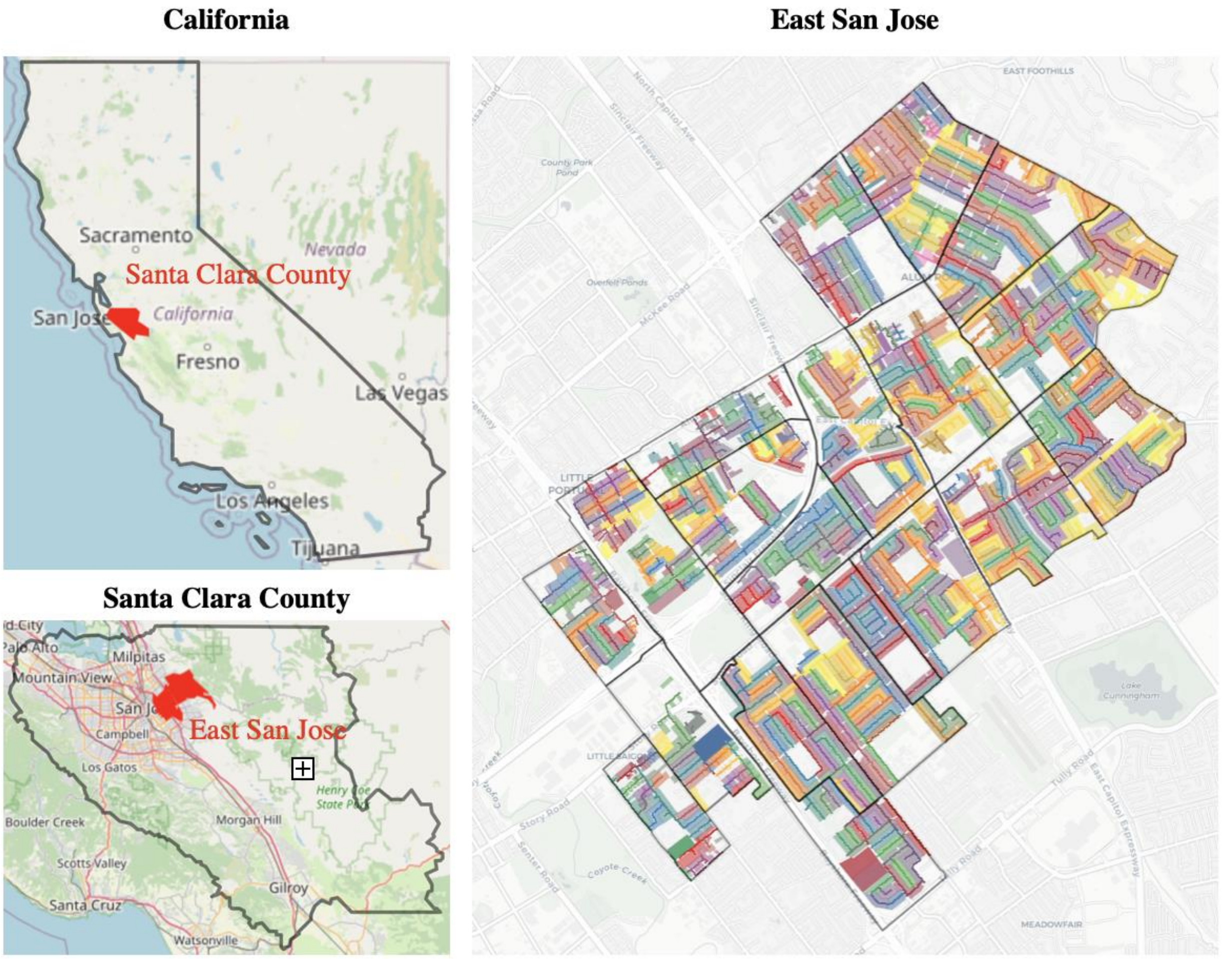

Upper left: State of California (outlined in black), with Santa Clara County shaded in red. Bottom left: Santa Clara County (outlined in black) with the three ZIP codes of interest shaded in red. Right: Census tracts (outlined in black) entirely within the three ZIP codes and the refinement of those tracts into “segments" (colored). One colored segment represents approximately 100 households. We focused on the census tracts contained entirely within the three ZIP codes due to resource constraints.

© 2021 Chugg B et al. *JAMA Health Forum.*

eFigure 4. Overview of the Workflow

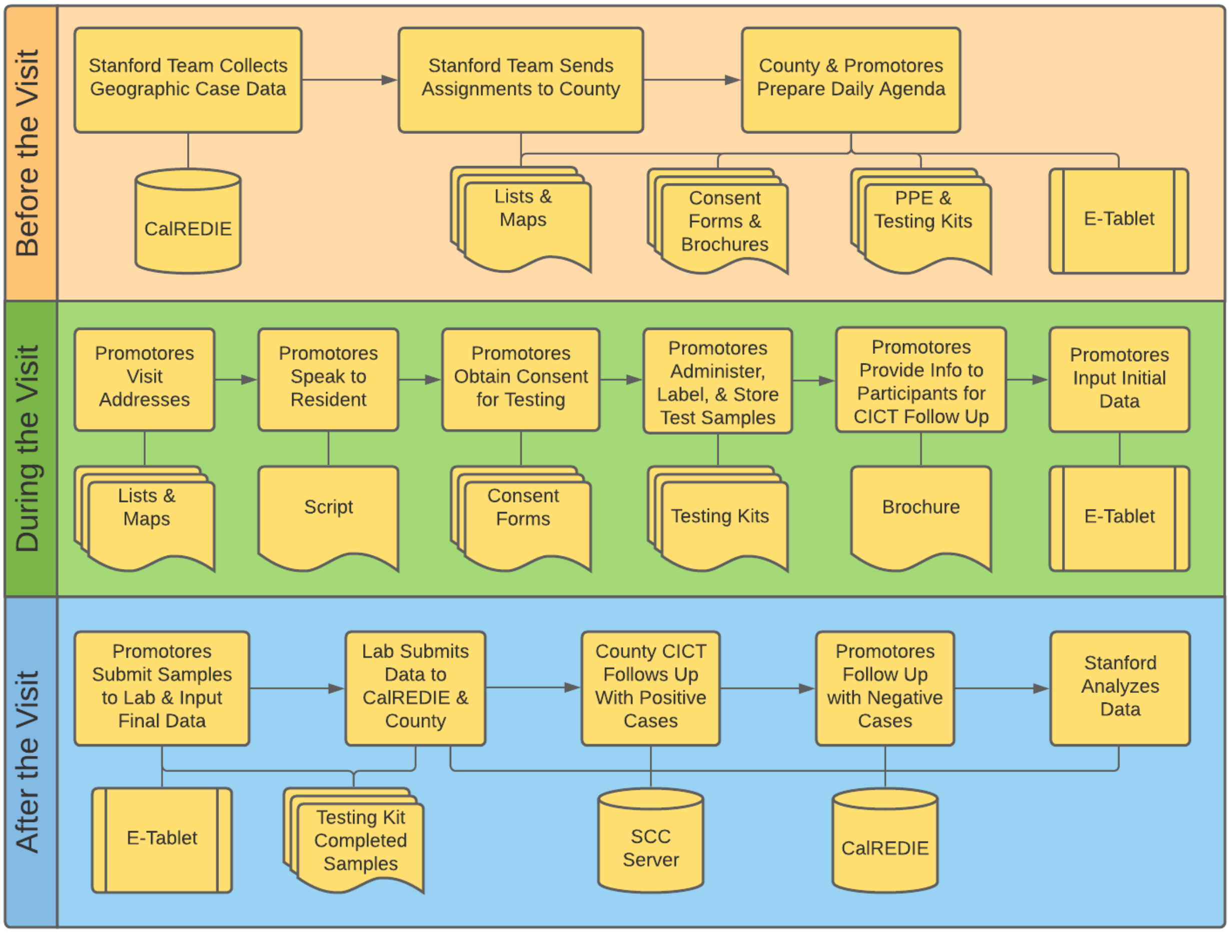

An overview of the workflow. We break the procedure into three phases: before the visit, during the visit, and after the visit. The steps occurring in the first phase occur the day prior to the visit, and involve segment selection by the Stanford team based on case rate data for that day. The selection occurs at approximately 4pm, after the CalREDIE data has been uploaded.

eTable. Demographic Breakdown of the 3 Door-to-Door Intervention Strategies

Demographic breakdown of the three door-to-door intervention strategies.

		**Uncertainty Sampling**	**Index Area Selection**	**Local Knowledge Selection**	***p*-value**
**Gender**	Female	59.6% [52.4, 66.5]	59.2% [51.4, 66.7]	62.7% [57.7, 67.5]	0.54
	Male	39.4% [32.5, 46.6]	39.6% [32.2, 47.4]	35.0% [30.2, 39.9]	
	Unknown / Other	1.0% [0.1, 3.6]	1.2% [0.1, 4.2]	2.3% [1.1, 4.4]	
**Ethnicity**	Latinx	77.8% [71.3, 83.4]	85.2% [78.9, 90.2]	93.6% [90.7, 95.8]	0.001
	Not Latinx	2.0% [0.6, 5.1]	3.6% [1.3, 7.6]	0.3% [0.0, 1.4]	
	Other / Declined	20.2% [14.8, 26.5]	11.2% [6.9, 17.0]	6.2% [4.0, 9.0]	
**Race**	African American	1.0% [0.0, 3.6]	0.5% [0.0, 3.2]	0.0% [0.0, 0.9]	0.001
	Asian	15.7% [10.9, 21.5]	10.1% [6,0, 15.6]	4.4% [2.6, 6.9]	
	White	23.7% [18.0, 30.3]	38.5% [31.1, 46.2]	24.7% [20.5, 29.3]	
	Multirace / Other	43.9% [36.9, 51.2]	47.9% [40.2, 55.7]	64.8% [59.8, 69.5]	
	Not Reported	15.7% [10.9, 21.5]	3.0% [1.0, 6.8]	6.2% [4.0, 9.0]	
**Age**	0-17 years	21.2% [15.7, 27.6]	21.9% [15.9, 28.9]	19.8% [15.9, 24.1]	0.02
	18-24 years	9.1% [5.5, 14.0]	8.9% [5.1, 14.2]	11.1% [8.1, 14.6]	
	25-44 years	30.3% [24.0, 37.2]	23.7% [17.5, 30.8]	28.3% [23.9, 33.0]	
	45-64 years	24.7% [18.9, 31.4]	26.0% [19.6, 33.3]	32.1% [27.5, 37.0]	
	65+ years	14.6% [10.0, 20.4]	19.5% [13.8, 26.3]	8.7% [6.1, 12.0]	
	N	198	169	389	

eTable 1: 95% binomial confidence intervals are provided beneath each percentage. *p*-values from a χ^2-test compare the equivalence of all three distributions.

© 2021 Chugg B et al. *JAMA Health Forum.*

eReferences

1. Lauer SA, Grantz KH, Bi Q, et al. The Incubation Period of Coronavirus Disease 2019 (COVID-19) From Publicly Reported Confirmed Cases: Estimation and Application. Ann Intern Med. 2020;172(9):577-582. doi:10.7326/M20-0504
2. Brown LD, Cai TT, DasGupta A. Interval Estimation for a Binomial Proportion. Stat Sci. 2001;16(2):101-117.

© 2021 Chugg B et al, *JAMA Health Forum*.
